# Human glycolipid transfer protein (*GLTP*) genes: organization, transcriptional status and evolution

**DOI:** 10.1186/1471-2164-9-72

**Published:** 2008-02-08

**Authors:** Xianqiong Zou, Taeowan Chung, Xin Lin, Margarita L Malakhova, Helen M Pike, Rhoderick E Brown

**Affiliations:** 1 The Hormel Institute, University of Minnesota, Austin, Minnesota 55912, USA; 2 Department of Biochemistry, Yeungnam University, Kyeongsan 712-749, Republic of Korea

## Abstract

**Background:**

Glycolipid transfer protein is the prototypical and founding member of the new GLTP superfamily distinguished by a novel conformational fold and glycolipid binding motif. The present investigation provides the first insights into the organization, transcriptional status, phylogenetic/evolutionary relationships of *GLTP *genes.

**Results:**

In human cells, single-copy *GLTP *genes were found in chromosomes 11 and 12. The gene at locus 11p15.1 exhibited several features of a potentially active retrogene, including a highly homologous (~94%), full-length coding sequence containing all key amino acid residues involved in glycolipid liganding. To establish the transcriptional activity of each human *GLTP *gene, *in silico *EST evaluations, RT-PCR amplifications of *GLTP *transcript(s), and methylation analyses of regulator CpG islands were performed using various human cells. Active transcription was found for 12q24.11 *GLTP *but 11p15.1 *GLTP *was transcriptionally silent. Heterologous expression and purification of the GLTP paralogs showed glycolipid intermembrane transfer activity only for 12q24.11 GLTP. Phylogenetic/evolutionary analyses indicated that the 5-exon/4-intron organizational pattern and encoded sequence of 12q24.11 *GLTP *were highly conserved in therian mammals and other vertebrates. Orthologs of the intronless *GLTP *gene were observed in primates but not in rodentiates, carnivorates, cetartiodactylates, or didelphimorphiates, consistent with recent evolutionary development.

**Conclusion:**

The results identify and characterize the gene responsible for GLTP expression in humans and provide the first evidence for the existence of a *GLTP *pseudogene, while demonstrating the rigorous approach needed to unequivocally distinguish transcriptionally-active retrogenes from silent pseudogenes. The results also rectify errors in the *Ensembl *database regarding the organizational structure of the actively transcribed *GLTP *gene in *Pan troglodytes *and establish the intronless *GLTP *as a primate-specific, processed pseudogene marker. A solid foundation has been established for future identification of hereditary defects in human *GLTP *genes.

## Background

Glycosphingolipids (GSLs) and related metabolites are found ubiquitously in eukaryotes and mediate key functions involving cell membranes, including immune responses, drug resistance, surface adhesion, neuroregeneration, differentiation, and apoptosis [1-6]. Glycolipid transfer protein (GLTP) is a soluble protein (209 amino acids; ~24 kDa) that can selectively transfer GSLs between membranes [7-13]. GLTP has been implicated in the Golgi-to-plasma membrane, nonvesicular trafficking of glucosylceramide, a key intermediate in the synthesis of higher GSLs and in cellular drug resistance [11,14,15]. In order to bind glycolipid, GLTP utilizes an all α-helix conformation, arranged in a two-layer 'sandwich motif' containing no intramolecular disulfides, to form a single glycolipid liganding site [16-18]. Comparative structural analyses of GLTP in various GSL-free and GSL-complexed forms, along with crystallographic B-factor distributions, suggest that a cleft-like gating mechanism, involving conformational changes to two interhelical loops and one α-helix, facilitates entry and exit of the lipid chains in the membrane-associated state. Acquisition of glycolipid occurs via an adaptive recognition process involving a sugar headgroup recognition center that forms multiple hydrogen bonds and van der Waals contacts to selectively anchor the sugar-amide moieties to amino acid side chains of the protein surface as well as a 'molded-to-fit', hydrophobic tunnel to accommodate the hydrocarbon chains of the ceramide moiety [13,16-18].

The novel architecture of GLTP markedly contrasts the conformational motifs of other lipid binding and transfer proteins which generally are dominated by β-sheet, i.e. β-grooves/concave cups and β-barrels, or helical bundles stabilized by multiple disulfide-bridges, i.e. saposin-folds. Such proteins include sphingolipid activator proteins, CD1 proteins, ceramide transfer protein, phosphoglyceride transfer proteins, other START-related proteins, nonspecific lipid transfer proteins, fatty acid binding proteins, lipocalins, and plant lipid transfer proteins [13,16,17]. GLTP also forms a membrane targeting motif that differs from those used by other important peripheral proteins, i.e., C1, C2, PH, FYVE, and PX domains. The unique structural aspects of the glycolipid binding pocket and the membrane interaction region of GLTP establish the GLTP-fold as the prototype of the GLTP superfamily [19-22]. GLTP domains recently have been identified in other proteins, such as the ubiquitously-expressed phosphatidylinositol 4-phosphate (PtdIns(4)P) adaptor protein-2 (FAPP2) which is involved in vesicular trafficking from the Golgi to the cell surface and glucosylceramide transfer during GSL synthesis [23,24]. Other GLTP orthologs have been linked to the programmed cell response induced by stress in plants (e.g. ACD11) [25] and as part of the self-nonself recognition and compatibility response during heterokaryon fusion in filamentous fungi (e.g. HET-C2) [13,26].

The present study provides the first characterization of genes encoding GLTP. In humans, single-copy *GLTP *genes were located to chromosomes 11 and 12, encoding highly homologous GLTPs (~94%). The *GLTP *gene at locus 11p15.1 exhibited several characteristic features of a potentially active retrogene, affording the opportunity to critically evaluate RT-PCR limitations for accurate determination of single-copy, pseudogene expression. Assessment of transcriptional activity by RT-PCR required extensive controls to ensure accurate conclusions and was aided by determination of the methylation status of 5' UTR CpG islands as well as by comparative phylogenetic/evolutionary analyses of human *GLTP *genes with other vertebrate *GLTP *genes. The organizational pattern of the transcribed human *GLTP *gene (5-exon/4-intron and exon sizes) and the encoded GLTP amino acid sequence were highly conserved among various therian mammals and other vertebrates. In contrast, orthologs of the human intronless *GLTP *gene were found only in primates (*Pan*, *Macaca*) and were not present in rodentia (*Mus*, *Rattus*) carnivora (*Canis*), cetartiodactyla (*Sus*, *Bos*), or didelphimorphia (*Didelphis*), consistent with recent evolutionary development as a primate-specific, processed pseudogene.

## Results

### Organization of Human *GLTP *Genes

**Figure 1:**
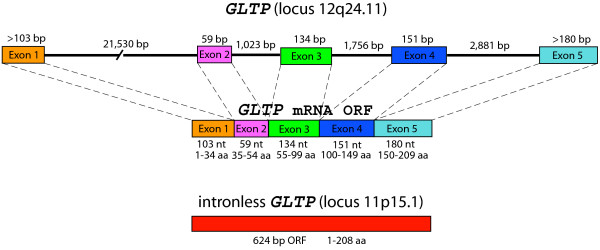
Organization of Human *GLTP *Genes.

To identify the human gene(s) encoding GLTP, the sequences of cDNAs that we previously cloned from *GLTP *mRNA of human skin fibroblasts and glioma cells (GenBank AF209074, AY372530, AY372531, AY372532) were used in BLAST searches of the NCBI Human Genome. Highly homologous genomic DNA sequences were found at loci on two different chromosomes: 12q24.11 of chromosome 12 (>99% identity) and 11p15.1 of chromosome 11 (~94% identity). Five separate sequences, interspersed at the 12q24.11 locus, were identical with five segments comprising the full-length *GLTP *cDNA ORF (630 bases), suggesting the presence of a *GLTP *gene with five exons separated by four introns (Figure 1). When adjoined in 5'-to-3' fashion, the five exons encoded the same 209 amino acid sequence that we previously determined for human GLTP by RT-PCR using purified mRNA [16,27]. The first intron was substantially larger (21,530 bases) than the other three introns (1,023, 1,756, and 2,881 bases). All exon/intron boundaries were characterized by classic consensus nucleotide sequences expected for splice sites, i.e. introns contained 5' GT and 3' AG dinucleotides as well as upstream pyrimidine tracts [28]. The 5'-3' arrangement of the five exons, the presence of classic intron dinucleotide sequences at all exon/intron junctions, and the 100% homology of the five exons at the 12q24.11 locus with the full-length ORF in *GLTP *mRNA were consistent with transcript maturation by *cis *splicing processes [28].

**Figure 2:**
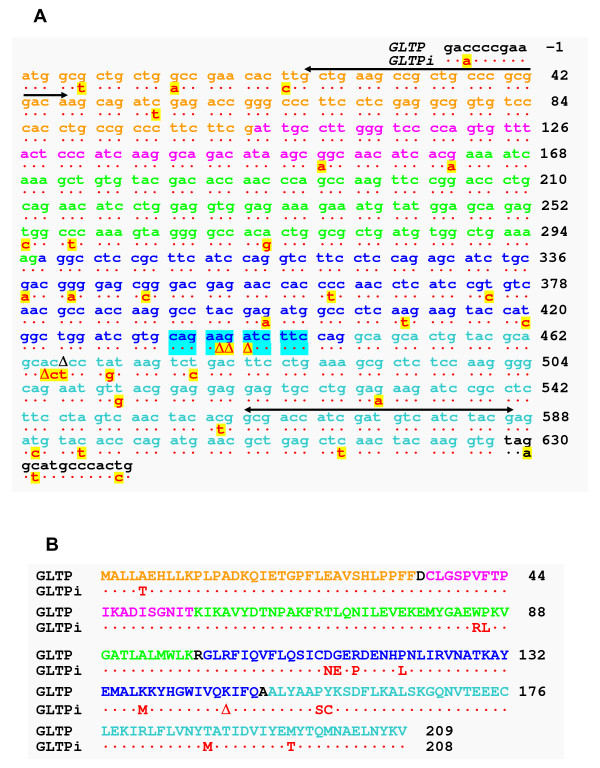
**Sequences of Human *GLTP *Genes and Predicted Translation Products**. **A**. Nucleotide ORF Sequences of 5-exon/4-intron *GLTP *and intronless *GLTP *(*GLTPi*). The black horizontal lines show the locations of the *GLTP *'universal primers' used for PCR analyses. **B**. Encoded Amino Acid Sequences of Human GLTP Homologs. Different sequence coloring distinguishes the exon source of 5-exon/4-intron *GLTP*.

Alignment of the human cDNA ORFs (630 bases) encoding GLTP (AF209074, AY372530, AY372531, AY372532) also revealed ~94% homology with a single genomic DNA sequence at locus 11p15.1 in chromosome 11 (Figure 2A). The genomic sequence (627 bases) contained both start (ATG) and stop (TAA) codons and encoded a near full-length GLTP-like protein (208 a.a.) lacking only Lys146 (Figure 2A). Absent from the *GLTP*-like sequence was AGA from within the CAG^435^AAGATC TTC^444 ^region, affecting two potential codons (Figure 2A; cyan highlight). Fortuitously, the deleterious effect of the missing AGA was minimized and resulted only in the loss of a single lysine residue. The remaining CAG^435^ATC TTC^441 ^sequence kept the reading frame fidelity in tact without alteration of subsequent codons. Altogether, nucleotide deletions and substitutions delineated a total of 13 different amino acids in the GLTP paralog, resulting in 94% identity with GLTP encoded at locus 12q24.11 (Figure 2B).

Comparison of the encoded sequences for human GLTP and the GLTP paralog (Figure 2B) revealed the presence of all residues previously shown to be important components of the glycolipid liganding site, encompassing both the glycolipid recognition center (Asp48, Asn52, Lys55, Trp96, His140, Tyr207, Val209) and the hydrophobic tunnel (Leu4, Leu30, Phe33, Phe34, Leu37, Val41, Phe42, Pro44, Ile45, Ile49, Phe103, Ile104, Phe107, Leu108, Ala128, Leu136, His140, Val144, Ile147, Ala151, Leu152, Ala155, Phe161, Leu165) [16,29]. The 94% overall homology of the GLTP paralog for human GLTP was similar to that of mouse GLTP and was much greater than that of HET-C2 fungal GLTP ortholog, which actively transfer glycolipids [26]. Thus, the evidence suggested that the intronless *GLTP *gene (*GLTPi*) at locus 11p15.1 might be a retrogene with the potential to express active protein.

### Transcriptional Status of Human *GLTP *Genes

To determine whether the NCBI human expressed sequence tag (EST) database contained any evidence for mRNA transcript of the 11p15.1 GLTP paralog, BLAST searches were performed against both the *GLTPi *ORF at 11p15.1 and the 5-exon/4-intron *GLTP *ORF at 12q24.11. More than 100 sequences matched positively to human 5-exon/4-intron *GLTP *ORF, indicating active transcription for the *GLTP *gene at locus 12q24.11 in a variety of cells. However, one EST clone (CF619013) derived from pooled chondrosarcoma tumor cells matched identically with the *GLTPi *ORF at locus 11p15.1, with its nearly full-length ORF (627/630 bases) including start (ATG) and stop (TAA) codons. Thus, the possibility remained that *GLTPi *at 11p15.1 could be an active retrogene, expressed only at certain times during cell development or only in select cell types [30]. To directly assess the transcriptional status of both *GLTP *genes *in vivo*, RT-PCR analyses were performed on the total RNA isolates from various human cells. To circumvent the difficult task of identifying primers and PCR cycling conditions that could reliably and unequivocally discriminate between the highly homologous ORFs predicted from the two *GLTP *genes, PCR reactions were performed with '*GLTP *universal primers' designed to amplify equally well either 562 or 559 base sequences of either *GLTP *ORF (Figure 2A). This strategy had the benefit of providing a PCR response expected to directly correlate with the relative abundance of the two potential *GLTP *transcripts, irrespective of chromosomal origin. Careful optimization of PCR cycling conditions avoided caveats associated with high cycling PCR, which can amplify the slightest traces of genomic DNA present in RNA isolates and lead to erroneous conclusions. Controls using only Platinum *Taq *DNA polymerase without reverse transcriptase (No-RT controls; see Additional file 1, Figure S1) established the critical number of PCR cycles in our experiments to be 35–37 to maximize detection of potential *GLTP *transcripts from the single-copy genes while avoiding spurious contributions from amplified genomic DNA of *GLTPi *remaining in the DNase-treated transcript isolates.

**Figure 3:**
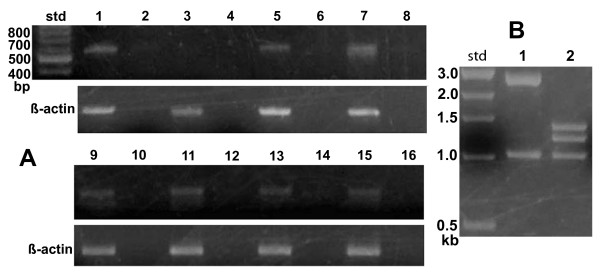
**Transcriptional Status of Human *GLTP *Genes**. **A**. Agarose gel electrophoresis patterns of PCR products (37 cycles) obtained with 'universal GLTP primers' from different cells. Odd numbered lanes = RT-PCR; Even numbered lanes = No-RT PCR controls (PCR with no reverse transcriptase); Lanes 1 & 2 = glioma cells; Lanes 3 & 4 = human skin fibroblasts; Lanes 5 & 6 = human HBL100 breast cancer cells; Lanes 7 & 8 = Gaucher cells; Lanes 9 & 10 = human T47D breast cancer cells; Lanes 11 & 12 = human IMR32 neuroblastoma cells; Lanes 13 & 14 = human HTB126 breast cancer cells; Lanes 15 & 16 = human Caov3 ovarian cancer cells. β-actin transcription is shown in subtending panel at 30 PCR cycles. **B**. Agarose gel electrophoresis patterns obtained after *Bsp*HI restriction digestion of pGEM-T clones containing *GLTP *ORFs originating from 5-exon/4-intron *GLTP *(Lane 1) or from intronless *GLTP *(Lane 2). Std = molecular weight standards.

Figure 3A shows the results obtained using an optimized number of PCR thermal cycles (37 cycles) with DNase-treated RNA isolates from glioma cells (lane 1), human skin fibroblasts (lane 3), HBL100 breast cancer (lane 5), T47D breast cancer (lane 9), breast cancer HTB-126 (lane 13), Gaucher cells (lane 7), Caov3 ovarian cancer (lane 15), IMR-32 neuroblastoma (lane 11). No-RT PCR controls are shown in even numbered lanes (Figure 3A). Cloning of PCR products using pGEM-T vector and restriction analysis using *Bsp*HI enabled efficient and rapid determination of RT-PCR product source (e.g., 5-exon/4-intron *GLTP *or *GLTPi*). Figure 3B shows the control *Bsp*HI restriction pattern obtained by digestion of pGEM-T vector with the different *GLTP *ORF inserts (corresponding pGEM-T vector map and *Bsp*HI sites, see Additional file 1, Figure S1A). Analyses of more than 120 clones from different cell lines (Figure 3A) using optimized PCR conditions produced restriction patterns consistent only with the 5-exon/4-intron *GLTP *gene being the source of transcribed mRNA (see Additional file 1, Figure S1C). Sequencing of representative clones verified the restriction digest patterns. Only when the number of RT-PCR cycles exceeded 40 were clones occasionally found displaying the *GLTPi *cDNA restriction pattern (see Additional file 1, Figure S1B). Even so, No-RT PCR controls were consistent with the *GLTPi *cDNA being derived from trace contamination by *GLTPi *genomic DNA. Thus, active transcription involved only the single-copy, 5-exon/4-intron *GLTP *gene, with transcript levels being relatively high in human glioma cells and Gaucher cells. Although expression levels in human skin fibroblasts were relatively low and difficult to visualize in agarose gels (Figure 3A, lane 3), the presence of 5-exon/4-intron *GLTP *PCR product was confirmed by excision and cloning of the corresponding gel region. Transcription of *GLTPi *was not evident in any of the analyzed cells by the preceding criteria.

### Methylation Status of CpG Islands in Human *GLTP *Genes

**Figure 4:**
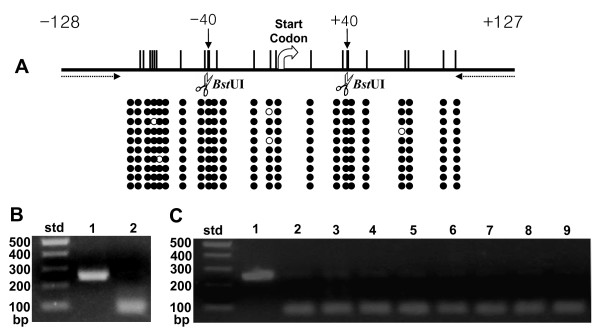
**Methylation Status of Human Intronless *GLTP *Gene**. Vertical bars show location of CpG dinucleotides. *Bst*UI restriction sites, consisting of adjacent CpG dinucleotides, are shown by scissors. Individual clones are shown by horizontal rows of circles. Filled circles represent methylated CpG dinucleotides; unfilled circles represent nonmethylated CpG dinucleotides. Primers for RT-PCR (255 base product) are indicated by dotted arrows. The ATG start codon represents nucleotides +1, +2, and +3. **B. & C**. Combined bisulfite restriction analysis (COBRA) of intronless *GLTP *gene methylation status. **B**. Agarose gel electrophoresis patterns obtained before (Lane 1) and after (Lane 2) *Bst*UI restriction digestion of bisulfite-treated PCR products for intronless *GLTP *gene (*GLTPi*) in human blood cell genomic DNA. **C**. COBRA analysis of intronless *GLTP *methylation status in different cell types. Lane 1 = pooled PCR products before *Bst*UI restriction digestion; Lanes 2–9 shows action of *Bst*UI restriction digestion. Lane 2 = glioma cells; Lane 3 = human skin fibroblasts; Lane 4 = human HBL100 breast cancer cells; Lane 5 = Gaucher cells; Lane 6 = human T47D breast cancer cells; Lane 7 = human IMR32 neuroblastoma cells; Lane 8 = human HTB126 breast cancer cells; Lane 9 = human Caov3 ovarian cancer cells. Because of the absence of *Bst*UI restriction sites in amplified PCR products of the CpG island of the 5-exon/4-intron region, COBRA analysis could be used to verify the absence of methylation detected by direct sequencing and served only as a negative control for *Bst*UI action shown Panel C. Std = molecular weight standards.

CpG dinucleotides in the 5' end regions of transcriptionally active chromosomal alleles generally are unmethylated. Conversely, methylation of CpG islands is a known indicator of transcriptional silencing [31-33]. In human *GLTPi*, a 255 base sequence including the 5' UTR (-128 bases) and initial portion (20%) of the potential ORF (see additional file 1, Figure S2), met the criteria of a CpG island [31-34]. To assess methylation status, human blood genomic DNA was treated with bisulfite to convert unmethylated cytosines to uracils while leaving any methylated cytosines unaltered. Primers containing no CpG dinucleotides were used for PCR to avoid bias between DNA templates based on their original methylation status [34,35]. The results obtained by sequencing, after clonal amplification using pGEM-T, are shown in Figure 4. The 23 CpG dinucleotides, localized in the CpG island of *GLTPi*, were highly methylated in all clones (Figure 4A, filled circles), consistent with transcriptional silencing.

In the 5-exon/4-intron *GLTP *gene, the CpG island (666 bases) included a larger 5' UTR region (-476 bases) along with all of the Exon 1 ORF (103 bases) and the initial 87 bases of Intron 1 (see Additional file 1, Figure S2). Consistent with designation as a CpG island was the very high G-C content (76% overall), that reached 80% and 84% in the 5'UTR regions -450 and -300 from the translation initiation site, respectively. The very high concentration of CpG dinucleotides (88 sites) in the CpG island severely limited the locations where 'CpG-free' primers, having the required 20–30 base sequence length, could be used to obtain the desired PCR products (<300 base long) [34,35]. The criteria were met by a 201 base sequence containing 11 CpG sites in the 5'UTR (-559 to -359) of the 5-exon/4-intron *GLTP *gene. Bisulfite treatment followed by sequencing of clones showed all 11 CpG sites to be completely unmethylated (see Additional file 1, Figure S2), consistent with active transcription of the 5-exon/4-intron *GLTP *gene.

To confirm the sequencing data for human blood cell genomic DNA and further analyze the methylation status of the CpG island of *GLTPi *in various other human cells, combined bisulfite-restriction analysis (COBRA) was performed (35). Figures 4B and 4C show the COBRA data obtained for the *GLTPi *CpG island. Figure 4B shows the PCR products after bisulfite treatment of human blood cell genomic DNA before (lane 1) and after (lane 2) *Bst*UI restriction digestion, which cuts only when two adjoining CpG dinucleotides within the recognition site are methylated (lane 2). Figure 4C shows results obtained with glioma (lane 2), skin fibroblasts (lane 3), breast cancer [HBL100 (lane 4), T47D (lane 6), and HTB-126 (lane 8)], Caov3 ovarian cancer (lane 9), IMR-32 neuroblastoma (lane 7) and Gaucher cells (lane 5). Although traces of undigested amplified products were detected (Figure 4C, lanes 2–9), nearly all amplified PCR products from *GLTPi *CpG islands were cut by B*st*UI, consistent with high level methylation and with transcriptional silencing.

### Glycolipid Transfer Activity of Human GLTP Homologs

**Figure 5:**
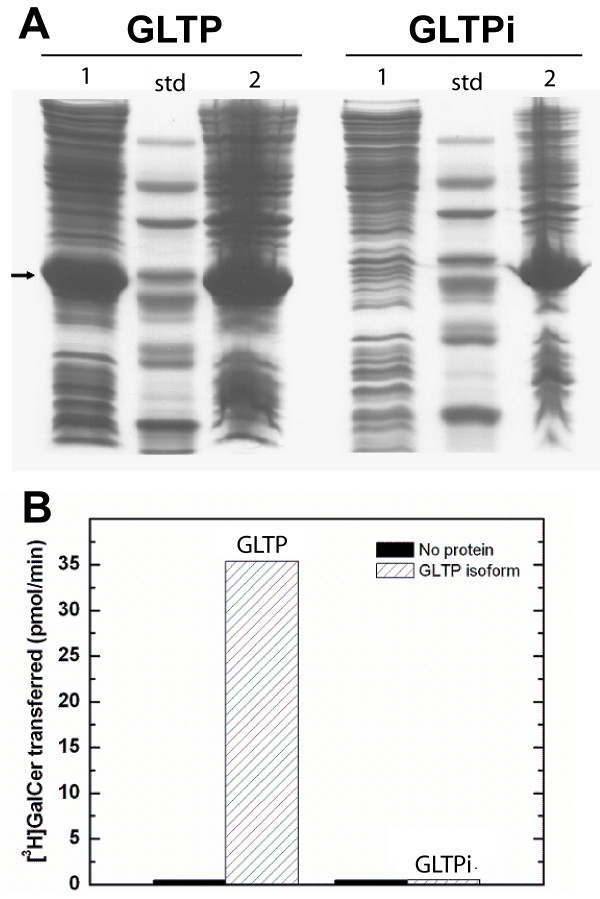
**Heterologous Expression and Glycolipid Intermembrane Transfer Activity of Human GLTP Homologs**. **A**. Heterologous expression of GLTP homologs in *E. coli *using pET-30 vector shown by SDS-PAGE. Arrow shows migration position of 6xHis-S-GLTP homologs (~25 kDa). GLTP = left panel; Intronless GLTP (GLTPi) = right panel; Lane 1 = soluble fraction; Lane 2 = Inclusion body pellet. Std = molecular weight standards. **B**. Intermembrane glycolipid transfer activity of GLTP homologs. [^3^H]GalCer transfer measured as described in Methods and Materials. The bars represent the averages of 6 assays performed using 1.0 μg of GLTP homolog.

As pointed out earlier, comparison of human 5-exon/4-intron *GLTP *with *GLTPi *revealed encoding of all amino acid residues known to be important components of the glycolipid liganding site, including the glycolipid recognition center (Asp48, Asn52, Lys55, Trp96, His140, Tyr207, Val209) and the hydrophobic tunnel (Leu4, Leu30, Phe33, Phe34, Leu37, Val41, Phe42, Pro44, Ile45, Ile49, Phe103, Ile104, Phe107, Leu108, Ala128, Leu136, His140, Val144, Ile147, Ala151, Leu152, Ala155, Phe161, Leu165) [16,29]. To directly test whether *GLTPi *could be a source of active protein in human cells, the two GLTP homologs were heterologously expressed in *E. coli*, purified from the soluble fractions, and analyzed for their ability to transfer glycolipid between phospholipid bilayer vesicles *in vitro*. Two findings were noteworthy. First, compared to 5-exon/4-intron GLTP, the solubility of the GLTPi paralog was very poor, as indicated by its high accumulation in the inclusion bodies of *E. coli *(Figure 5A), consistent with a tendency to misfold. Second, GLTPi paralog purified from the soluble fraction showed negligible activity compared to purified 5-exon/4-intron GLTP (Figure 5B) when assessed for glycolipid intermembrane transfer using the well-established radiolabeled and fluorescence energy transfer assays [27,29,36,37]. Inclusion of Triton X-100 at low concentration during protein expression did result in slightly increased distribution of GLTPi paralog to *E. coli *soluble fractions. Nonetheless, the resulting GLTPi paralog (purified from the soluble fraction) remained inactive (data not shown).

To determine whether a mammalian cellular environment is needed for expression of active GLTPi paralog, human embryonic kidney cells (HEK 293T) were transfected with FLAG-CMV vector containing the *GLTPi *and *GLTP *ORFs. Analysis of the resulting cell supernatant fractions revealed a 20-fold increase in glycolipid intermembrane transfer activity above endogenous background levels for cells transfected with *GLTP*, but no increase in glycolipid intermembrane transfer activity for cells transfected with *GLTPi *(data not shown).

### Evolutionary Insights

Table 1 summarizes the structural patterns found in the multi-exon *GLTP *genes of various vertebrates. The 5-exon/4-intron organizational pattern, characteristic of the human *GLTP *gene at locus 12q24.11, was absolutely conserved, as were the sizes of analogous exons (e.g. exon 1 always 103 bases; exon 2 always 59 bases, etc.) in representative therian mammals and other vertebrates. In contrast, the size of analogous introns varied considerably among the 5-exon/4-intron *GLTP *genes. Nonetheless, intron 1 was consistently much larger than the other three introns and was exceptionally large in primates (~21,000 bases).

**Table 1:** Orthologs of Human 5-exon/4-intron *GLTP *Gene in Different Vertebrates

Organism (GenBank ID)	Exon 1 ORF	Intron 1	Exon 2	Intron 2	Exon 3	Intron 3	Exon 4	Intron 4	Exon 5 ORF	Identity^a^
*Homo sapiens *(NM_016433)	103	21,530	59	1,023	134	1,756	151	2,881	183	100
*Pan troglodytes *(EF688398)	103	N.D.^b^	59	1,023	134	1,754	151	N.D.^b^	183	100
*Macaca mulatta *(XM_001089145)	103	21,127	59	1,032	134	1,773	151	3,017	183	100
*Canis familaris *(XM_849301)	103	14,621	59	996	134	2,697	151	1,674	183	99
*Sus scrofa *(NM_213822)	103	N.D.^b^	59	N.D.^b^	134	N.D.^b^	151	N.D.^b^	183	98
*Bos taurus *(NM_175799)	103	14,693	59	957	134	2,776	151	2,233	183	98
*Mus musculus *(NM_019821)	103	13,079	59	1,222	134	2,060	151	3,464	183	94
*Rattus norvegicus *(XM_213793)	103	13,396	59	1,668	134	1,882	151	1,558	183	93
*Monodelphis domestica *(XM_001364000.1)	103	7,138	59	1,873	134	3,442	151	408	183	89
*Xenopus tropicalis *(NM_001016039.2)	103	21,680	59	373	134	201	151	3,371	183	74
*Danio rerio *(Chromosome 5) (XM_681171)	103	5,545	59	83	134	3,039	151	809	183	79
*Danio rerio *(Chromosome 10) (XM_692094.1)	103	1,808	59	1,772	134	2,216	151	1,331	183	71

^a^The percent identity is compared to the human protein sequence. ^b^Not yet determined.

In certain cases, sequence data for *GLTP *genes was found to be either missing or of very low reliability. This situation was evident for the predicted *GLTP *gene of *Pan troglodytes *(GenBank XM_522526; Ensembl, ver. 46, August 2007; [38]), based on careful comparisons to the human and macaque genes. To rectify the issue, the problematic G-C rich region of the chimp *GLTP *gene (e.g. exon 1) was cloned and sequenced using genomic DNA from chimpanzee fibroblasts (GenBank EF520721) by including PCR enhancers known to facilitate cloning of *GLTP *mRNA [27,36]. The resulting sequence of exon 1 in the chimp *GLTP *gene was found to be identical to that of the human 5-exon/4-intron *GLTP *gene (see Additional file 1, Figure S3). Molecular cloning of the *GLTP *ORF using mRNA from chimp fibroblasts (GenBank EF688398) confirmed the identical nature of the encoded chimp and human GLTPs (209 a.a.).

Alignment of the five *GLTP *exons comprising the primate mRNA ORFs revealed very high conservation of nucleotide sequence (see Additional file 1, Figures S3 and S4). The human and chimp ORFs were 99.5% identical, i.e. 627/630 bases, and the human and macaque ORFs were 97.5% identical, i.e. 614/630 bases. All nucleotide differences were synonymous [39,40], i.e. no altered codon translation and all three primate GLTPs were identical in amino acid sequence (see Additional file 1, Figure S5). In nonprimate mammals, the *GLTP *ORFs had more nonsynonymous base substitutions (see Additional file 1, Figure S4), resulting in increased amino acid variability (see Additional file 1, Figure S5). Even so, high amino acid sequence homology was encoded in the 5-exon/4-intron *GLTP *genes of nonprimate mammals with sequence identity being 99% for Canis, 98.6% for Bos, 94.3% for Mus, and 90.4% for Monodelphis as well as for other vertebrates (e.g., 74.2% for *Xenopus*, 80.4% and 72.2% for *Danio*) compared to human 5-exon/4-intron *GLTP*.

**Figure 6:**
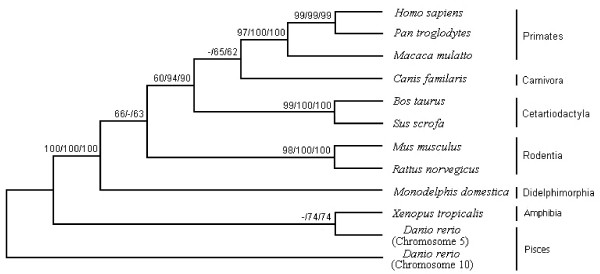
**Phylogenetic Tree of 5-exon/4-intron *GLTP *Gene**. Numbers correspond to percentages of bootstrap support for each node from the maximum parsimony, distance, and minimum evolution analyses. (Nucleotide and amino acid sequence alignments are provided as Additional file 1, Figures S4 and S5).

Phylogenetic analyses (Figure 6) indicated that all sequences produced exactly the same tree topology by three independent methods [neighbor-joining (NJ), maximum parsimony (MP) and minimum evolution (ME)] [39,40]. Most of the major internal branches were well supported (Figure 6). Overall, the phylogenetic/evolutionary relationships observed for the 5-exon/4-intron *GLTP *gene conformed to the widely-accepted phylogeny of vertebrates. The situation for *GLTPi *was much different. Orthologs of the human *GLTPi *gene were discovered only in nonhuman primates (chimpanzee, macaque) and were not present in carnivora (dog), cetartiodactyla (pig, cow), rodentia (mouse, rat) or didelphimorphia (opossum), suggesting recent evolutionary development. The 'intronless' nature of the ORF sequences was consistent with origination by retrotransposition of mRNA derived from an ancestral 5-exon/4-intron *GLTP *gene. The structural features supporting the common evolutionary ancestry of *GLTPi *genes in primates, included the following differences compared to their 5-exon/4-intron *GLTP *genes: 1) location on a different chromosome; 2) occurrence of nearly full-length *GLTP *ORFs (624 bases) including both start and stop codons; 3) absence of AGA at the same location in the sequence; 4) presence of the CAG^435^ATC TTC^441 ^sequence (after AGA deletion from the CAG^435^AAGATC TTC^444 ^region), keeping the potential reading frame fidelity intact and limiting downstream changes. The locations of the base changes within the affected *GLTPi *sequences are shown in Additional file 1, Figure S3. These shared and delineating features provided an unequivocal indication of the close evolutionary relationship among the *GLTPi *genes of primates.

Estimates of the evolutionary divergence for the 5-exon/4-intron and *GLTPi *genes of primates and other primates provided insights into the selection pressure exerted on different phylogenetic branches. As shown in Table 2, base substitutions resulting in no change in amino acid residue (*K*s) per synonymous site are compared with the nucleotide changes that do result in an amino acid alteration per nonsynonymous site (*K*a) [41,42]. *K*_a_/*K*_s _ratios indicate the rate of evolution [42-44]. In Table 2, the higher *K*_a_/*K*_s _ratios for 5-exon/4-intron *GLTP *genes among rodents compared to those among primates and cetartiodactylates are consistent with the rate of 5-exon/4-intron GLTP protein evolution being faster in rodents than in primates and cetartiodactyla, after scaling to neutral divergence. Nonetheless, very low values for the *K*_a_/*K*_s _ratios, i.e. zero and < 0.01 for the exons of the 5-exon/4-intron *GLTP *genes in primates (*Homo sapiens*, *Pan troglodytes*, and *Macaca mulatta*) and cetartiodactyla (*Bos Taurus and Sus scrofa*) and of *Mus musculus *and *Rattus norvegicus*, are consistent with strong selection pressure to maintain and conserve the encoded amino acid sequence, as expected for genes playing important functional roles. In contrast, the *GLTPi *genes of *Homo sapiens*, *Pan troglodytes*, and *Macaca mulatta *show significantly higher *K*_a_/*K*_s _values (Table 2), consistent with no evolutionary selection pressure being exerted on the primate *GLTPi *compared to their 5-exon/4-intron *GLTP *genes, because *K*a/*K*s values slightly below 1.0 are not *de facto *evidence of purifying selection pressure [45].

**Table 2:** Synonymous (*K*_s_) and nonsynonymous (*K*_a_) nucleotide substitution rate for ORFs of vertebrate *GLTP *genes

Species^a^	Substitutions	bp^b^	*K*_s_	*K*_a_	*K*_a_/*K*_s_
***GLTP***					
Hom/Pan	3	627	0.01895	0.00000	0.00000
Hom/Mac	17	627	0.10175	0.00000	0.00000
Pan/Mac	16	627	0.09212	0.00000	0.00000
Bos/Sus	8	627	0.04131	0.00000	0.00000
Rat/Mus	33	627	0.22413	0.00196	0.00874
Dan(chr5)/(chr10)^c^	194	627	1.99122	0.19415	0.09750
***GLTPi***					
Hom/Pan	12	624	0.02959	0.01556	0.52585
Hom/Mac	44	624	0.08889	0.06576	0.73979
Pan/Mac	43	624	0.09884	0.05994	0.60643

^a^*GLTP *= functional 5-exon/4-intron *GLTP *gene and *GLTPi *= intronless *GLTP *gene. Hom, Pan, Mac, Bos, Sus, Mus, Rat and Dan indicate *Homo sapiens*, *Pan troglodytes*, *Macaca mulatta*, *Bos taurus*, *Sus scrofa*, *Mus musculus*, *Rattus norvegicus *and *Danio rerio*, respectively. ^b^Number of base pairs compared. ^c^The relatively high *K*_a_/*K*_s _ratio of 5-exon/4-intron *GLTP *genes between *Danio rerio GLTP *genes on chromosome 5 and chromosome 10 suggests a functional change after duplication of one of the *Danio rerio GLTP *genes.

## Discussion

GLTP has recently emerged as the prototypical and founding member of a new protein superfamily, characterized by a unique conformational fold for lipid binding/transfer as well as for protein targeting/translocation to membranes [13,17,19-22]. While recent advances have delineated the architecture used by GLTP to selectively acquire glycosphingolipid ligand [16-18] as well as the role played by membrane lipid composition in regulating GLTP activity *in vitro *[13,37,46-50], the organization, transcriptional status, phylogenetic/evolutionary relationships of human *GLTP *genes have not been previously characterized.

Our findings show the *GLTP *gene to be present in all vertebrate genomes and characterized by a highly conserved 5-exon/4-intron organizational pattern, consistent with mRNA production via classical *cis *processing events. The resulting *GLTP *transcript encodes a highly conserved mammalian protein able to selectively bind glycolipids and accelerate their intermembrane transfer [13]. The human 5-exon/4-intron *GLTP *gene maps to locus 12q24.11 of chromosome 12 and is actively transcribed in variety of different cells, as shown by RT-PCR and by the unmethylated status of CpG dinucleotides in its CpG island. Noteworthy features of the 5-exon/4-intron *GLTP *gene are the very large size of its first intron (>21,000 bases) and the exceptionally high density of CpG dinucleotides (88) and of G-C bases (~80%) in its CpG island. The very high G-C content probably contributed to the poor sequence resolution evident in the *GLTP *gene of the recent *Pan troglodytes *(chimpanzee) genome release (GenBank NW_001223167; Sept. 2006), the resulting omission of exon 1 from the chimp *GLTP *gene (GenBank XM_522526), and inaccurate predictions of mRNA and protein sequence (Ensembl version 46, August 2007; [38]). Our cloning and sequencing of the genomic DNA of *GLTP *exon 1 as well as full-length *GLTP *ORF from mRNA of *Pan troglodytes *fibroblasts (GenBank EF520721 &EF688398) have rectified the earlier inconsistencies and demonstrated the existence of the highly conserved 5-exon/4-intron *GLTP *gene in chimpanzee.

The discovery of a second, intronless *GLTP*-like gene (*GLTPi*), encoding a full-length GLTP paralog in humans provided the opportunity to test for functionality as a retrogene. The issue is of timely relevance because of recent reports indicating transcription and regulatory functions of intronless pseudogenes [51-56]. Our analyses of *GLTPi*, which maps to locus 11p15.1 of chromosome 11, provide a clear indication of the challenges associated with definitive validation of pseudogene transcription. The experiments illustrate how PCR amplification involving closely-related sequences can detect even the slightest traces of genomic DNA contaminant in highly purified RNA isolates (see Additional file 1, Figure S1). It is clear that stringent controls are needed when using PCR to analyze the transcriptional status of intronless genes, including PCR controls performed in the absence of reverse transcriptase (No-RT controls), to avoid erroneous conclusions regarding the source of similarly sized templates (e.g. mRNA ORF versus genomic DNA of intronless gene). Such controls are needed even with DNase-treated mRNA isolates (see Additional file 1, Figure S1). In fact, it was possible to detect *GLTPi *genomic DNA in No-RT controls of mRNA isolates treated three times in succession with DNase, when the PCR thermal cycling number reached 42 or higher (data not shown). The PCR-associated caveats emphasize the need for independent analyses during evaluations of the transcriptional status of intronless genes that are highly homologous to the transcript of their parent genes. Reliance on the EST database as a complementary approach is risky because of documented evidence of substantial contamination from various sources including genomic DNA [57-59]. In contrast, a dependable complementary approach for monitoring gene transcriptional status is analysis of the methylation status of CpG islands commonly found in the 5'UTR of genes. The high methylation levels of the 23 CpG dinucleotides in the CpG island of the intronless *GLTP*-like gene, along with the carefully controlled PCR data, provide strong evidence for this gene being a transcriptionally silent pseudogene. We find this to be the case despite the fact that *GLTPi *encodes an almost full-length ORF (627 vs 630 bases), complete with start and stop codons, and with an overall amino acid sequence homology of 94% compared to the active 5-exon/4-intron GLTP. It is noteworthy that the 94% homology is similar to that of human 5-exon/4-intron GLTP and mouse GLTP and much greater than that of the HET-C2 fungal ortholog [26] which actively transfers glycolipid. Also, heterologous expression of 5-exon/4-intron GLTP cDNA generated from human, bovine, porcine, and murine transcripts yields fully active protein in *E. coli *[27,29,36,47-50], presumably because GLTP lacks intramolecular disulfides [13,16] and needs no posttranslational modifications, i.e. glycosylation or acylation [8,27]. Nonetheless, the human GLTPi paralog is inactive and poorly soluble when expressed in *E. coli*. Ectopic expression in human embryonic kidney cells using FLAG-CMV vector also resulted in inactive GLTPi. The absence of *in vitro *activity in the human GLTPi paralog occurs despite the fact that contains all residues previously identified as important components of the glycolipid liganding site, including the glycolipid recognition center (Asp48, Asn52, Lys55, Trp96, His140, Tyr207, Val209) and the hydrophobic tunnel (Leu4, Leu30, Phe33, Phe34, Leu37, Val41, Phe42, Pro44, Ile45, Ile49, Phe103, Ile104, Phe107, Leu108, Ala128, Leu136, His140, Val144, Ile147, Ala151, Leu152, Ala155, Phe161, Leu165) [16,29]. Our findings suggest that misfolding, triggered by one or more of the 13 altered residues, is responsible for the inactive state of human GLTPi. Identification of which of these GLTP residues or combination of residues are essential for proper folding will require future in-depth analyses.

Additional support for human *GLTPi *gene being a transcriptionally silent pseudogene comes from phylogenetic/evolutionary divergence analyses that provide insights into the selection pressure being exerted on genes [41-44]. The frequencies of nonsynonymous (*K*a) and synonymous (*K*s) nucleotide substitutions for the *GLTP *functional and intronless genes (Table 2) are consistent with strong divergence of *GLTPi *relative to the 5-exon/4-intron *GLTP *genes. The relatively high *K*a/*K*s ratios (> 0.5) for *GLTPi *of *Homo sapiens*, *Pan troglodytes*, and *Macaca mulatta *indicate that they have been subjected to very low evolutionary selection pressure. It is important to recognize that *K*a/*K*s values slightly below 1.0 are not *de facto *evidence of significant purifying selection pressure. Indeed, a comprehensive genome-wide survey of human pseudogenes, performed against benchmark control sets of true genes and pseudogenes, revealed that only a small fraction (~12%) of pseudogenes have *K*a/*K*s values very close to the theoretical neutral selection pressure value of 1.0 [45]. Rather, the majority of human pseudogenes have *K*a/*K*s values very similar to those of the primate *GLTPi *pseudogenes. In contrast, the low *K*a/*K*s ratios for the 5-exon/4-intron *GTLP *genes (e.g., 0 to <0.01) indicate strong purifying selection pressure to conserve the encoded amino acid sequence in order to maintain protein functionality.

## Conclusion

The present study provides the first insights into the genomic organization, transcriptional status, and phylogenetic/evolutionary relationships of human *GLTP *genes, which encode the prototypical member of the recently established GLTP superfamily. The finding of a highly conserved 5-exon/4-intron *GLTP *gene in vertebrates vouches for the importance of GLTP in cells. Also noteworthy was the discovery of many errors in the on-line bioinformatics databases pertaining to *GLTP *genes, perpetuated by predictive modeling analyses that relied on low stringency survey data (e.g. exon deletion in the chimp *GLTP *gene) rather than on carefully executed experiments. The novel finding of a transcriptionally-silent, intronless *GLTP *gene in humans supports designation as *GLTPP1*, in accordance with guidelines for human gene nomenclature [60,61]. The discovery of *GLTPP1 *orthologs in other primates provides a new 'selection pressure free' marker for tracking primate evolutionary relationships. Clear delineation of the absence of transcription by human *GLTPP1 *was evident only by application of comprehensive and stringent experimental criteria, thus highlighting the challenges of unequivocally distinguishing silenced pseudogenes from functional retrogenes. In a larger context, the present study supports concerns about the validity of recent functional roles assigned to intronless pseudogenes. Among the most noteworthy is the recent re-evaluation of *Mkrn1-p1 *pseudogene 'functionality' [62], providing definitive evidence that the *Mkrn1-p1 *pseudogene is neither expressed nor imprinted, nor does it regulate its source gene in trans, despite earlier high profile indications to the contrary [54]. Equally important, the fundamental insights gained here into human *GLTP *gene organization, transcriptional status, and evolution provide a firm foundation for future elucidation and understanding of hereditary defects involving *GLTP *genes.

## Methods

### Sequence Data

Sequences determined as part of this study and deposited with the GenBank Data Library are: AF209704, AY372530, AY372531, AY372532, AF209703, AY039109, EF520721, and EF688398

### Cell Culture and Genomic DNA Extraction

All human cell lines were supplied by the American Type Culture Collection cell bank (ATCC, Rockville, MD). Human glioma cells, skin fibroblasts, HEK 293T cells, Gaucher cells, CaOV3 ovarian cancer cells, and breast cancer cells (HBL100, T47D, HTB-126) were cultured at 37°C, 5% CO_2_, in Dulbecco modified Eagle medium (DMEM) (Mediatech Inc, Herndon, VA) supplemented with 10% heat-inactivated fetal bovine serum (Innovative Research, MI). Human IMR32 neuroblastoma cells were cultured similarly but in DMEM supplemented with 10% newborn calf serum (GIBCOBRL, Grand Island NY). Chimpanzee skin fibroblasts (Coriell Institute, Camden, NJ) were cultured at 37°C, 5% CO_2_, in MEM Alpha Modified medium with nucleosides and L-glutamine (Fisher Sci., USA) supplemented with 10% heat-inactivated fetal bovine serum (Innovative Research, MI).

### RT-PCR and DNA Sequencing

Total RNA was isolated from cultured cells using Trizol reagent (GBICOL/BRL, Gaithersburg, MD, USA) according to the manufacturer's instructions, and was treated with DNase I (Sigma-Aldrich, St. Louis, MO) at 25°C for 15 min to minimize contamination by genomic DNA. SuperScript One-Step RT-PCR kits (Invitrogen, Carlsbad, CA) with Platinum *Taq *were used with RNA template (1 μg). Human GLTP-specific primer pairs were 5'TGAAGCCGCTGCCCGCGGACA3' and 5'CGTAGATGACATCGATGGTCG3'. RT-PCR reaction thermocycling conditions were: 30 min at 50°C, 2 min at 94°C or pre-cycle and then different cycles of 15 sec at 94°C, 30 sec at 63°C, 1 min at 72°C. β-actin served as internal RT-PCR control, performed in parallel with *GLTP *RT-PCR, using β-actin primer pairs of 5'GGCATCCTCACCCTGAAGTA3' and 5'CCATCTCTTGCTCGAAGTCC3' to produce a 496 base PCR fragment. With β-actin, PCR was limited to 30 cycles to avoid nonlinear responses. No-RT analysis also was performed as a control for detection of genomic DNA. PCR products were analyzed by 1.2% agarose gel electrophoresis, purified using a gel purification kit (NucleoTrap^® ^Gel Extraction Kit, Clontech, Mountain View, CA), and cloned using pGEM-T vector (Promega, Madison, WI, USA). Plasmids were digested by BspHI restriction enzyme, and the products were analyzed by agarose gel electrophoresis. Representative clones were sequenced by automated dideoxynucleotide sequencing using an ABI Prism 3730xl DNA analyzer (GENEWIZ, South Plainfield, NJ, USA).

### DNA Methylation Analysis

Genomic DNA from human blood (Promega) or isolated from various cell lines (Sigma-Aldrich, GenElute™ mammalian genomic DNA miniprep kit) was bisulfite converted using manufacturer's recommendations (CpGenome Fast; Chemicon, Temecula, CA). Primers to amplify 5' regions of *GLTP *and intronless *GLTP *genes were (5'AATAGGAGAGTAGT-GTTTATTTAGGTTATT3' & 5'ATCTTCCCCAAATATACCTCCC3') and (5'TTATTGTGATTATGA-AAATATATTAA-AAA3' & 5'TAAACACTAAAAACCCAAAACAATC3'), respectively, using Platinum^® ^Taq DNA Polymerase (Invitrogen, Carlsbad, CA). Cycling conditions for intronless *GLTP *were: step1, 94°C for 3 min; step 2, 94°C for 30 sec; step 3, 50°C for 30 sec; step 4, 72°C for 30 sec; step 5, repeat 36 times; and step 6, extend at 72°C for 10 min. For *GLTP*, cycling conditions were the same except: step 2, 90°C for 30 sec and step 3, 55°C for 30 sec. Amplified products were separated on 1.2% agarose gels, and the 255 bp (intonless *GLTP*) and 201 bp (*GLTP*) bands were excised. The purified products from human blood genomic DNA were cloned directly into pGEM-T for DNA sequence analysis (Genewiz, South Plainfield, NJ).

COBRA analysis was performed as described previously [35]. The sodium bisulfite modified PCR products from different cell lines were reamplified for 38 PCR cycles using high-fidelity polymerase (*Taq *Plus, Stratagene, La Jolla, CA). PCR products (15 μl) were restriction digested for 4 h at 60°C by addition of a master mix (10 μl) equivalent to New England Biolabs Buffer #2 (2.5 μl), water (5.5 μl) and *Bst*UI (2 μl of 10 Units/μl; New England Biolabs). Restriction digests was analyzed by 1.2% agarose gel electrophoresis and ethidium bromide staining.

### Plasmid Construction and GLTP Expression

*GLTP *and *GLTPi *ORFs were inserted into pET-30 Xa/LIC vector (Novagen) by ligation independent cloning [29]. Positive clones were verified for reading frame fidelity by nucleotide sequencing prior to transforming BL21(DE3) cells. For 6xHis-S-GLTP expression, bacterial cultures were grown at 37°C in Luria-Bertani medium (750 ml) until reaching OD_600 _= 0.9–1.1. Cells were then induced by 0.1 mM isopropyl-beta-D-galactopyranoside and bacterial growth was continued at 15°C for 20 h. The cell pellet was resuspended in washing buffer (30 ml) containing 10 mM imidazole, 150 mM NaCl, 50 mM NaH_2_PO_4_, pH 8.0, 10% glycerol, 1 mg/ml lysozyme and 10 mM β-mercaptoethanol. After brief sonication and centrifugation, clarified cell lysate was loaded on 1–1.5 ml Ni-NTA affinity resin (Qiagen). The majority of protein was soluble. Protein was released from the washed column by stepwise elution with buffer containing increasing concentrations of imidazole (60, 100 and 200 mM) in 150 mM NaCl, 50 mM NaH_2_PO_4_, pH 8.0, and 5% glycerol. Imidazole was removed from combined fractions by desalting columns (Econo-Pac 10 DG; Bio-Rad) equilibrated with 150 mM NaCl and 50 mM NaH_2_PO_4_, pH 8.0 and protein fractions were concentrated to 2–6 mg/ml using Centriplus centrifugical filter devices YM-10 (Amicon). Glycerol was added at 10% final concentration to stabilize and protect from freeze-thaw damage. Protein purity was analyzed by 15% SDS-PAGE and Coomassie staining. Protein concentration was measured by the Bradford assay (BioRad Protein Assay) using bovine serum albumin as standard. 6xHis-S-tag was removed from rGLTP by incubation with Factor Xa (Novagen) for 16 h at room temperature to obtain a sequence identical to wild-type GLTP. The protein was purified by FPLC SEC using a HiLoad 16/60 Superdex-75 prep grade column (Amersham) equilibrated with 150 mM NaCl, 20 mM Tris (pH 8.0). Protein fractions were concentrated to 2–6 mg/ml and stored in buffer containing 10% glycerol.

HEK 293T cells were plated at 1 × 10^5 ^densities, incubated until reaching 40–70% confluency, and transfected with pFLAG-CMV™-4 vector (2 μg) using jet PEI (Polypus Transfection, New York NY), a cationic polymer polyethylenimine (PEI) that forms complexes with DNA for efficient transfection of mammalian cells, after insertion of the wild-type human *GLTP *and *GLTPi *ORFs using the *Bam*HI (5') and *Eco*RI (3') restriction sites and ligating with T4 ligase overnight at 16°C. Control cells were transfected with pFLAG-CMV™-4 vector containing no GLTP cDNA insert. After 36 h, transfected cells were selected based on resistance to G418 sulfate (400 μg/ml). Cells were harvested by scraping into buffer containing 20 mM Tris, 50 mM sucrose, 2 mM EDTA, 2 mM DTT, aprotinin, leupeptin, and PMSF. Homogenization was performed by 20 strokes with a Dounce homogenizer. The supernatant was recovered by centrifugation at 80 g for 10 min and assayed for glycolipid transfer activity.

### Assay of Glycolipid Transfer Protein Activity

Glycolipid transfer activity was monitored as described previously [29,37]. Briefly, donor vesicles comprised of 89 mol% 1-palmitoyl-2-oleoyl phosphatidylcholine (POPC), 1 mol% [^3^H]GalCer, 10 mol% negatively charged dipalmitoyl phosphatidic acid and traces of [^14^C]tripalmitate (nonexchangeable marker) were prepared by sonication in 10 mM sodium phosphate (pH 7.4), 1 mM DTT, 1 mM EDTA, and 0.02% NaN_3_. Sonicated POPC vesicles served as acceptor membranes. After incubation with GLTP (0.5 – 1 μg) for 30 min at 37°C, charged donor and neutral acceptor vesicles were separated by rapid elution over DEAE Sephacel minicolumns and the glycolipid transfer activity of GLTP was determined by liquid scintillation counting of the eluants.

### Bioinformatics Analyses

*GLTP *genes were identified by searches of NCBI databases [63] and ENSEMBL [38] against GLTP ORFs that we previously cloned from different mammalian cells [16,27,36,47]. BLAST searches of the nononredundant and EST databases [64] also were performed. K-Estimator 6.0, a divergence estimator program employing the Kimura two-parameter correction method for multiple hits, was used to estimate the frequency of synonymous and nonsynonymous nucleotide substitutions [41]. Phylogenetic analyses were carried out using three different methods, i.e. neighbor-joining (NJ), maximum parsimony (MP) and minimum evolution (ME), to construct distance trees with MEGA (Version 4.0) [39,40]. Bootstrap values were calculated from 1000 replicates and values of <50% are not shown. CLUSTLX analysis [65] was used to comparatively align the open reading frames of the vertebrate *GLTP *genes.

## Abbreviations

GLTP, glycolipid transfer protein; GSL, glycosphingolipid; EST, expressed sequence tag; RT-PCR, reverse transcription polymerase chain reaction; POPC, 1-palmitoyl-2-oleoy-*sn*-glycero-3-phosphocholine.

## Authors' contributions

XZ and TC performed the RT-PCR transcriptional analyses. XZ performed the methylation and phylogenetic/evolutionary analyses. TC and XL cloned *GLTP *cDNAs from various cells and TC cloned the human *GLTP *pseudogene. MLM, TC and HMP expressed, purified, and analyzed the glycolipid transfer activity of the human GLTP paralogs. XZ, TC, and REB participated in the design and execution of the study. REB wrote and constructed the manuscript with contributions from XZ and TC. All authors read and approved the final manuscript.

## Supplementary Material

Additional file 1 Figure S1: RT-PCR/Restriction Analyses of Human *GLTP *and *GLTPi *cDNA in Various Human Cells. The strategy for distinguishing human *GLTP *and *GLTPi *cDNA clones by *Bsp*HI restriction analysis after RT-PCR is depicted. In 3xDNase-treated RNA isolates, *GLTPi *cDNA clones occasionally are detected using 42 PCR cycles (panel B) but not when using 37 or fewer PCR cycles (panel C). The data suggest that genomic DNA is virtually impossible to completely eliminate from RNA isolates. Independent assessment using No-RT PCR (e.g. Figure 3A, even numbered lanes) is required to clearly distinguish the source template (gDNA versus mRNA).
Figure S2: CpG Islands in Human *GLTP *genes. The figure shows CpG islands in human *GLTP *genes, identified using MethPrimer [34], and primer locations for COBRA analyses. The 5' untranslated region of human intronless *GLTP *gene (locus 11p15.1, chromosome 11) was found to be highly methylated (Figure 4), consistent with transcriptional silencing.
Table S1: Number of synonymous substitutions per site (*K*_s_), non-synonymous substitutions per site (*K*_a_) and confidence intervals. Divergence analyses based on estimates of the frequency of nonsynonymous (*K*_a_) and synonymous (*K*_s_) nucleotide substitutions between *GLTP *functional genes and intronless genes reveal that the intronless *GLTP *genes have diverged relative to 5-extron-4 intron *GLTP*. The data support information in Table 2 and suggest that the 5-extron-4-intron *GLTP *genes have been subjected to strong selection pressure to conserve their amino acid sequences, characteristic of functional genes. The intronless *GLTP *genes are under much less selection pressure compared to the 5-extron-4-intron *GLTP *genes.
Figure S3: *GLTP *and *GLTPi *Nucleotide Sequence Conservation in Primates. The data show the aligned nucleotide sequences and exon organizations for the *GLTP *genes and intronless *GLTPi *genes of humans, chimpanzees, and macaques.
Figure S4: *GLTP *ORF Conservation in Vertebrates. The data show the aligned nucleotide sequences for the *GLTP *open reading frames found in various vertebrates from humans to fish.
Figure S5: Conservation of GLTP Amino Acid Sequence in Vertebrates. The data show the aligned amino acid sequences for GLTPs that occur in various vertebrates from humans to fish. Click here for file
