# Supplementary material for: Human glycolipid transfer protein (GLTP) genes: organization, transcriptional status and evolution
Source: BMC Genomics. 2008 Feb 8;9:72. doi: 10.1186/1471-2164-9-72 (PMC2262070; doi:10.1186/1471-2164-9-72)
Supplement: Additional file 1 — Figure S1: RT-PCR/Restriction Analyses of Human GLTP and GLTPi cDNA in Various Human Cells. The strategy for distinguishing human GLTP and GLTPi cDNA clones by BspHI restriction analysis after RT-PCR is depicted. In 3xDNase-treated RNA isolates, GLTPi cDNA clones occasionally are detected using 42 PCR cycles (panel B) but not when using 37 or fewer PCR cycles (panel C). The data suggest that genomic DNA is virtually impossible to completely eliminate from RNA isolates. Independent assessment using No-RT PCR (e.g. Figure 3A, even numbered lanes) is required to clearly distinguish the source template (gDNA versus mRNA). Figure S2: CpG Islands in Human GLTP genes. The figure shows CpG islands in human GLTP genes, identified using MethPrimer [34], and primer locations for COBRA analyses. The 5' untranslated region of human intronless GLTP gene (locus 11p15.1, chromosome 11) was found to be highly methylated (Figure 4), consistent with transcriptional silencing. Table S1: Number of synonymous substitutions per site (Ks), non-synonymous substitutions per site (Ka) and confidence intervals. Divergence analyses based on estimates of the frequency of nonsynonymous (Ka) and synonymous (Ks) nucleotide substitutions between GLTP functional genes and intronless genes reveal that the intronless GLTP genes have diverged relative to 5-extron-4 intron GLTP. The data support information in Table 2 and suggest that the 5-extron-4-intron GLTP genes have been subjected to strong selection pressure to conserve their amino acid sequences, characteristic of functional genes. The intronless GLTP genes are under much less selection pressure compared to the 5-extron-4-intron GLTP genes. Figure S3: GLTP and GLTPi Nucleotide Sequence Conservation in Primates. The data show the aligned nucleotide sequences and exon organizations for the GLTP genes and intronless GLTPi genes of humans, chimpanzees, and macaques. Figure S4: GLTP ORF Conservation in Vertebrates. The data show the aligned nucleotide [file 1471-2164-9-72-S1.pdf]

Additional File #1

Human glycolipid transfer protein (*GLTP*) genes: organization, transcriptional status and evolution

Xianqiong Zou[1]*, Taeowan Chung[2]*, Xin Lin[1], Margarita L. Malakhova[1], Helen M. Pike[1], and Rhoderick E. Brown[1§]

[1]The Hormel Institute, University of Minnesota, Austin, Minnesota 55912, USA

[2]Department of Biochemistry, Yeungnam University, Kyeongsan 712-749, Republic of Korea

*These authors contributed equally to this work

[§]Corresponding author

Email addresses:

XZ: xzou@hi.umn.edu

TC: twchung@yu.ac.kr

XL: xinlin12342004@yahoo.com

MLM: margarita@hi.umn.edu

HMP: hmpike@hi.umn.edu

REB: reb@umn.edu

Figure S1 –RT-PCR/Restriction Analyses of Human *GLTP* and *GLTPi* cDNA in Various Human Cells

Different GLTP clones, produced by 37 PCR cycles and cloned using pGEM-T, were digested by *Bsp*HI and analyzed by agarose gel electrophoresis. The top panel (**A**) shows the *Bsp*HI restriction map for the different *GLTP* ORFs in pGEM-T. The middle (**B**) right panel shows the restriction analysis of various clones in which the RNA extracts were 3xDNase treated prior to RT-PCR (42 PCR cycles). Two clones containing the intronless *GLTP* ORF are clearly evident. No-RT PCR analysis revealed genomic DNA to be the source of the two clones [middle (**B**) left panel]. Similar occurrences of clones from the intronless *GLTP* ORF were evident in the absence of DNase I treatment after 37 PCR cycles. The lower (**C**) panel shows restriction analyses of various clones in which the RNA extracts were treated with DNase I. Intronless *GLTP* ORF was not detectable among the clones when the RT-PCR cycle number was 37 or less.

A

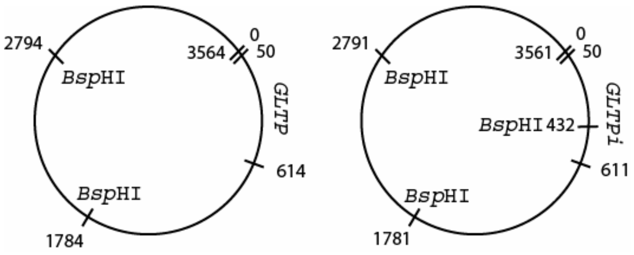

B

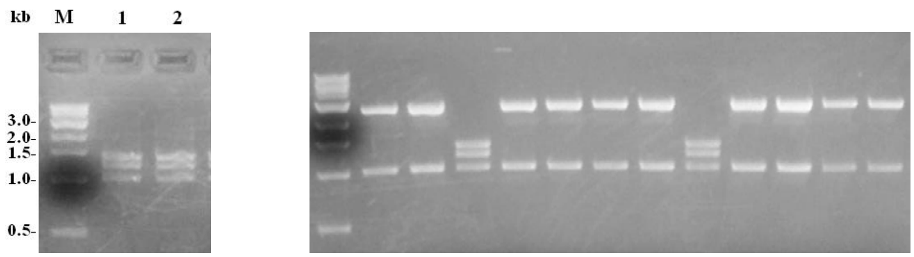

C

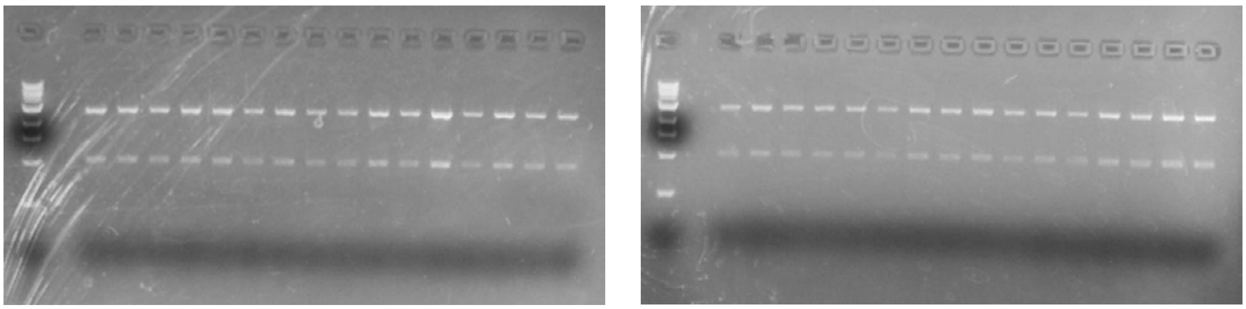

Figure S2 -CpG Islands in Human *GLTP* genes

Human *GLTPi* (intronless) --- locus 11p15.1 --- CpG Island

```
TCACTGTGATCATGAAAACATACCAAAAAAAGGGCTGTGACTGCCCGGGCGCTGACGGCGGCGGCG
GGGGGTGCGGTGTGGACGCCCGCGGTGCCGGCTGGGGCATCACCGTGGGCCTCGAACCCGAAATGG
CTCTGCTGACCGAACACCTGCTGAAGCCGTTGCCCGCGGACAAGCAGATCGAGACTGGGCCCTTCC
TCGAGGCGGTGTCCCACCTGCCGCCCTTCTTCGATTGCCTTGGGTCCCCAGTGTTTA
```

Predicted genomic sequence for fully methylated CpG sites

```
TTATTGTGATTATGAAAATATATTAAAAAAAGGGTTGTGATTGTTCGGGCGTTGACGGCGGCGGCG
GGGGGTGCGGTGTGGACGTTCGCGGTGTCGGTTGGGGTATTATCGTGGGTTTCGAATTCGAAATGG
TTTTGTTGATCGAATATTTGTTGAAGTCGTTGTTCGCGGATAAGTAGATCGAGATTGGGTTTTTTT
TCGAGGCGGTGTTTTATTTGTCGTTTTTTTTCGATTGTTTTGGGTTTTTAGTGTTTA
```

CpG island (yellow highlighted sequence), identified by MethPrimer [34] consisted of -119 bases upstream of ATG translation start site along with the initial 97 ORF bases. COBRA analysis involved PCR amplification of the 255 base sequence using primers delineated by the double underlined sequences.

***GLTP* (5-exon/4-intron)---locus 12q24.11 --- CpG Island**

```
-1000gaggatgttacttggtgggagaggatgaatgtgaagttacaaagtataaatggagaggatgtttct
tggagtgtgtggtctgtgtaaatggagaggatgaatgtgaagttacaaagtgtaaagggcgtgaatggaga
ggatgaagtgaagttgcaaagccgtttacattcctgtcattgctaaggtgcttttgatttaggtctagga
agtcagccttatgttccctgcctccaggccctatttttctgcctcacaacaggtaacatcagactgttgag
ggcagactggaccccagatttcgggaagcaggagttgtccagcagggcagtggctgagttagactgtgact
tcagcagtgttcaacaggtataaggtcattgaagaccaagaatcaagcaatcgagcgtcctcgtcggttgt
agcgaaagagaaccgtgaccaacaggagagcagtgtccacccaggtcacttggggttactggggtacaatt
ttgggggaaacggggtcctggggagcgttggctgggtggggctgtaacccgagctggtgggcggttgtg
agctgtgacctggcccgagtcggcccgcccggattgggggccaaaccggggcggcgggaggcacacctg
gggaagactcacctgggagcggctcacttgggccctccgggacccatgaaccggcgcacctcctctgcgtc
ccgccccgccggccccccggccaatgggggcgcggagacctcggcggcgggcgcggattgggagccggt
gagcgggggcggggcggcgcgcccggcagtttaggcgctcggccggcgggcggcgctgagcgctcggctac
gagggcgccgtgcgggcggaagtggcgacgctggcgtcccCGGAGCCTGCGGCGGGCGGCTGTGACGGCCC
CGGCGCTGACGGCGGCGGCCGGGGGGCGGTGTCGACGCCCGCGGCGCCGGCTGGGGCATCACCGCGGGCC
TCGACCCCGAAATGGCGCTGCTGGCCGAACACTTGCTGAAGCCGCTGCCCGCGGACAAGCAGATCGAGACC
GGGCCCTTCCTCGAGGCGGTGTCCCACCTGCCGCCCTTCTTCGgtgagccggaatggagggggaggcggc
ccgcagccccacgcgcacccgaatcctccttccccgagcatcccctaaccatctctgccctagacacccc
```

CpG island (yellow highlighted sequence), identified by MethPrimer [34] consisted of -476 bases adjacent to ATG translation start site, all of the Exon 1 ORF and a portion of Intron 1. 70 CpGs occur between -460 & 0 and 18 CpGs occur in the Exon 1 ORF + Intron region. Further upstream (-1000 & -500), only 9 CpGs occur. COBRA analysis, using primers delineated by the double underlined sequences, involved PCR amplification of the intervening 201 base sequence containing 11 CpGs.

Table SI

Number of synonymous substitutions per site (K_s), non-synonymous substitutions per site (K_a) and confidence intervals

			C. I.[a]									
			P=0.05		P=0.01			P=0.05		P=0.01	
Species[b]	No. bp[c]	K_s	Min.	Max.	Min.	Max.	K_a	Min.	Max.	Min.	Max.
GLTP											
Hom/Pan	627	0.0190	0.0000	0.0434	0.0000	0.0570	0.0000	0.0000	0.0000	0.0000	0.0000
Hom/Mac	627	0.1018	0.0566	0.1559	0.0446	0.1733	0.0000	0.0000	0.0000	0.0000	0.0000
Pan/Mac	627	0.0921	0.0497	0.1434	0.0391	0.1629	0.0000	0.0000	0.0000	0.0000	0.0000
Bos/Sus	627	0.0413	0.0098	0.0862	0.0048	0.1046	0.0000	0.0000	0.0000	0.0000	0.0000
Rat/Mus	627	0.2241	0.1466	0.3166	0.1213	0.3526	0.0020	0.0000	0.0082	0.0000	0.0116
Dan(chr5) /(chr10)	627	1.9912	1.4206	2.8613	1.2447	3.3960	0.1942	0.1402	0.2492	0.1240	0.2723
GLTPi											
Hs-Pan	624	0.0296	0.0049	0.0639	0.0000	0.0746	0.0156	0.0047	0.0295	0.0023	0.0345
Hs-Mac	624	0.0889	0.0419	0.1341	0.0310	0.1498	0.0658	0.0411	0.0939	0.0336	0.1039
Pan-Mac	624	0.0988	0.0487	0.1556	0.0394	0.1792	0.0599	0.0385	0.0876	0.0304	0.0992

[a]1000 replicates for confidence intervals (*C.I.*) [b] Hom, Pan, Mac, Bos, Sus, Mus, Rat and Dan indicate *Homo sapiens*, *Pan troglodytes*, *Macaca mulatta, Bos taurus*, *Sus scrofa*, *Mus musculus*, *Rattus norvegicus* and *Danio rerio*, respectively. *GLTP* = functional 5-exon/4-intron *GLTP* genes and *GLTPi* = intronless *GLTP* genes. [c]Number of base pairs compared.

FIGURE S3 -- *GLTP* and *GLTPi Nucleotide Sequence* Conservation in Primates. The nucleotide sequences are derived from the ORFs of the 5-exon/4-intron and intronless *GLTP* genes.

```
Hs GLTP     ATGGCGCTGCTGGCCGAACACTTGCTGAAGCCGCTGCCCGCGGACAAGCAGATCGAGACCGGGCCCTTCCTCGAGGCGGTGTCCCACCTGCCGCCCTTCTTCGAT 105
Hs GLTPi    .....T......A........C.....................................T............................................. 105
Pan GLTP    .....G......G........T.....................................C............................................. 105
Pan GLTPi   .....T...............C...........T.........................T............................................. 105
Mac GLTP    .....G.......................A...C.........................C................................A............ 105
Mac GLTPi   ..............T..............G.................T...........TA...A.......C................................ 105

Hs GLTP     TGCCTTGGGTCCCCAGTGTTTACTCCCATCAAGGCAGACATAAGCGGCAACATCACGAAAATCAAAGCTGTGTACGACACCAACCCAGCCAAGTTCCGGACCCTG 210
Hs GLTPi    .............................................A..........A................................................ 210
Pan GLTP    .............................................G..........G................................................ 210
Pan GLTPi   .............................................A..........A................................................ 210
Mac GLTP    .............................................G..........G...........C.....T.............................. 210
Mac GLTPi   .A..................................A.....................................C.....................T........ 210

Hs GLTP     CAGAACATCCTGGAGGTGGAGAAAGAAATGTATGGAGCAGAGTGGCCCAAAGTAGGGGCCACACTGGCGCTGATGTGGCTGAAAAGAGGCCTCCGCTTCATCCAG 315
Hs GLTPi    ..........................................C...T...............G.......................................... 315
Pan GLTP    ..........................................T...C...............A....................G..................... 315
Pan GLTPi   ..........................................C...................G....................A..................... 315
Mac GLTP    ..........................................T...................A.......................................... 315
Mac GLTPi   .......................................................A............AA........................A.......... 315

Hs GLTP     GTCTTCCTCCAGAGCATCTGCGACGGGGAGCGGGACGAGAACCACCCCAACCTCATCCGTGTCAACGCCACCAAGGCCTACGAGATGGCCCTCAAGAAGTACCAT 420
Hs GLTPi    .....................A...A.....C..............T............C.......................A..........T.........C 420
Pan GLTP    .....................G...G.....G..............C............T.......................G..........A.......... 420
Pan GLTPi   .....................A...A..............................T..C.......................A..........T.......... 420
Mac GLTP    .....................G...G..............................C..........................G..........A.......... 420
Mac GLTPi   ................C...T...................................................................................T 420

Hs GLTP     GGCTGGATCGTGCAGAAGATCTTCCAGGCAGCACTGTACGCAGCACCCTATAAGTCTGACTTCCTGAAAGCGCTCTCCAAGGGGCAGAATGTTACGGAGGAGGAG 525
Hs GLTPi    ................ΔΔΔ.........................CT...G......C...................................G............ 522
Pan GLTP    ................AGA.........................AC...A......T................................................ 525
Pan GLTPi   ................ΔΔΔ.................C.......CT...G......C.....G.......................................... 522
Mac GLTP    ................AGA.................T........C...A...A........C........A.......................A......... 525
Mac GLTPi   .........A......ΔΔΔ..................................G......A..........G................................. 522

Hs GLTP     TGCCTGGAGAAGATCCGCCTCTTCCTAGTCAACTACACGGCGACCATCGATGTCATCTACGAGATGTACACCCAGATGAACGCTGAGCTTAACTACAAGGTGTAG 630
Hs GLTPi    ........A............................T..........................C...T...................................A 627
Pan GLTP    ........G............................C..........................T...C...................................G 630
Pan GLTPi   .....................................T..........................G........................A..............A 627
Mac GLTP    .....................................C...C.................T....T...............T..C.....T..............G 630
Mac GLTPi   ...............................................................................TG........................ 627
```

FIGURE S4 -*GLTP* ORF Conservation in Vertebrates. The nucleotide sequences are derived from the ORFs of the 5-exon/4-intron *GLTP* genes.

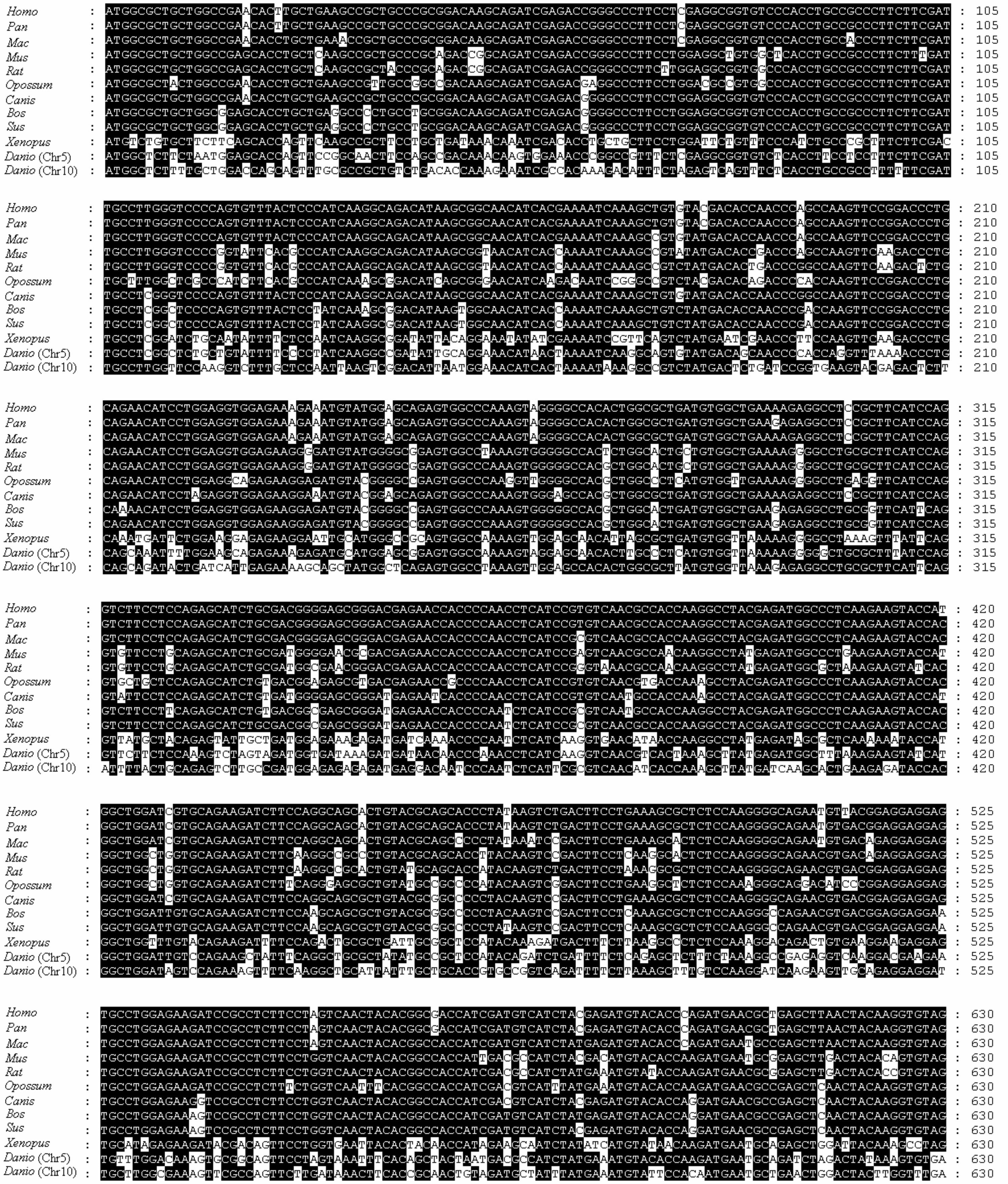

FIGURE S5 - Conservation of GLTP Amino Acid Sequence in Vertebrates.

The amino acid sequences are derived from the ORFs of the 5-exon/4-intron *GLTP* genes. CLUSTAL FORMAT for T-COFFEE Version_1.41, CPU=11.20 sec, SCORE=69, Nseq=9, Len=209

```
Homo             MALLAEHLLKPLPADKQIETGPFLEAVSHLPPFFDCLGSPVFTPIKADISGNITKIKAVY  60
Pan              MALLAEHLLKPLPADKQIETGPFLEAVSHLPPFFDCLGSPVFTPIKADISGNITKIKAVY  60
Macaca           MALLAEHLLKPLPADKQIETGPFLEAVSHLPPFFDCLGSPVFTPIKADISGNITKIKAVY  60
Canis            MALLAEHLLKPLPADKQIETGPFLEAVSHLPPFFDCLGSPVFTPIKADISGNITKIKAVY  60
Bos              MALLAEHLLRPLPADKQIETGPFLEAVSHLPPFFDCLGSPVFTPIKADISGNITKIKAVY  60
Mus              MALLAEHLLKPLPADRQIETGPFLEAVAHLPPFFDCLGSPVFTPIKADISGNITKIKAVY  60
Monodelphis      MALLAEHLLKPLPADKQIETRPFLDAVAHLPPFFDCFGSPIFTPIKADISGNIKTIRAVY  60
Xenopus          MSVLLQHQFKPLPADKQIDTCCFLDSVSHLPAFFDCLGSAIFSPIKADITGNISKIRSVY  60
Danio (chr.5)    MALLMEHQFRQLPADKQVETRPFLEAVSHLPPFFDCLGSAVFSPIKADIAGNITKIKAVY  60
Danio (chr.10)   MALLLDQQFAPLSDTKEIATKTFLESVSHLPPFFDCLGSKVFAPIKSDINGNITKIKAVY  60
                 *::* :: :  *.  ::: *  **::*:***.****:** :*:***:** ***..*::**

Homo             DTNPAKFRTLQNILEVEKEMYGAEWPKVGATLALMWLKRGLRFIQVFLQSICDGERDENH 120
Pan              DTNPAKFRTLQNILEVEKEMYGAEWPKVGATLALMWLKRGLRFIQVFLQSICDGERDENH 120
Macaca           DTNPAKFRTLQNILEVEKEMYGAEWPKVGATLALMWLKRGLRFIQVFLQSICDGERDENH 120
Canis            DTNPAKFRTLQNILEVEKEMYGAEWPKVGATLALMWLKRGLRFIQVFLQSICDGERDENH 120
Bos              DTNPTKFRTLQNILEVEKEMYGAEWPKVGATLALMWLKRGLRFIQVFLQSICDGERDENH 120
Mus              DTDPAKFKTLQNILEVEKGMYGAEWPKVGATLALLWLKRGLRFIQVFLQSICDGERDENH 120
Monodelphis      DTDPTKFRTLQNILEAEKEMYGAEWPKVGATLALMWLKRGLRFIQVLLQSICDGERDENR 120
Xenopus          ESNPSKFKTLQMILEGEKELHGPQWPKVGATLALMWLKRGLKFIQVMLQSIADGERDDQN 120
Danio (chr.5)    DSNPTRFKTLQQILEAEKEMHGAEWPKVGATLALMWLKRGLRFIQVLLQSLVDGDKDDNN 120
Danio (chr.10)   DSDPVKYETLQQILIIEKSSYGSEWPKVGATLALMWLKRGLRFIQILLQSLADGERDEDN 120
                 :::* ::.*** **  **  :*.:**********:******:***::***: **::*::.

Homo             PNLIRVNATKAYEMALKKYHGWIVQKIFQAALYAAPYKSDFLKALSKGQNVTEEECLEKI 180
Pan              PNLIRVNATKAYEMALKKYHGWIVQKIFQAALYAAPYKSDFLKALSKGQNVTEEECLEKI 180
Macaca           PNLIRVNATKAYEMALKKYHGWIVQKIFQAALYAAPYKSDFLKALSKGQNVTEEECLEKI 180
Canis            PNLIRVNATKAYEMALKKYHGWIVQKIFQAALYAAPYKSDFLKALSKGQNVTEEECLEKV 180
Bos              PNLIRVNATKAYEMALKKYHGWIVQKIFQAALYAAPYKSDFLKALSKGQNVTEEECLEKI 180
Mus              PNLIRVNANKAYEMALKKYHGWLVQKIFKAALYAAPYKSDFLKALSKGQNVTEEECLEKI 180
Monodelphis      PNLIRVNVTKAYEMALKKYHGWLVQKIFQGALYAAPYKSDFLKALSKGQDIPEEECLEKI 180
Xenopus          PNLIKVNITKAYEIALKKYHGWFVQKIFQTALIAAPYKDDFLKALSKGQTVKEEECIEKI 180
Danio (chr.5)    PNLIKVNVTKAYEMALKKYHGWIVQKLFQAALYAAPYRSDFLRALSKGREVKDEECLDKV 180
Danio (chr.10)   PNLIRVNITKAYDQALKRYHGWIVQKVFKAALFAAPCRSDFLKALSKDQEVAEEDCLAKV 180
                 ****:** .***: ***:****:***:*: ** *** :.***:****.: : :*:*: *:

Homo             RLFLVNYTATIDVIYEMYTQMNAELNYKV 209
Pan              RLFLVNYTATIDVIYEMYTQMNAELNYKV 209
Macaca           RLFLVNYTATIDVIYEMYTQMNAELNYKV 209
Canis            RLFLVNYTATIDVIYEMYTRMNAELNYKV 209
Bos              RLFLVNYTATIDVIYEMYTRMNAELNYKV 209
Mus              RLFLVNYTATIDAIYDMYTKMNAELDYTV 209
Monodelphis      RLFLVNFTATIDVIYEMYTKMNAELNYKV 209
Xenopus          RQFLVNYTTTIEAIYIMYNKMNAELDYKA 209
Danio (chr.5)    RQFLVNFTATNDAIYEMYTKMNADLDYKV 209
Danio (chr.10)   RQFLINFTATVDAIYEMYSTMNAELDYLV 209
                 * **:*:*:* :.** **. ***:*:* .
```
